# Genome-wide development of simple sequence repeats markers and genetic diversity analysis of chayote

**DOI:** 10.1186/s12870-024-05317-9

**Published:** 2024-06-26

**Authors:** Shaobo Cheng, Lihong Su, Xin Guo, Dalong Shao, Yanmei Qin, Xuanxuan Liu, Qianwen Chu, Xiaoting Zhou, Zhongqun He

**Affiliations:** College of Horticulture, Sichuan Agricultural University, Chengdu, 611130 PR China

**Keywords:** Chayote, SSR markers, Genetic diversity, Genome-wide, Association analysis

## Abstract

**Background:**

Chayote is a high economic crop in the Cucurbitaceae family, playing an important role in food production, disease treatment and the production of degradable materials in industries. Due to the harsh environment, such as high temperature, drought and frost, some chayote resources are gradually disappearing. It is crucial to collect, characterize, and conserve chayote resources. However, the genetic diversity of chayote resources in China has not been studied so far.

**Results:**

In this study, we collected 35 individuals of chayote from 14 provinces in China. Subsequently, we found 363,156 SSR motifs from the chayote genome and designed 57 pairs of SSR primers for validation. Out of these, 48 primer pairs successfully amplified bands, with 42 of them showing polymorphism. These 42 primer pairs detected a total of 153 alleles, averaging 3.64 alleles per locus. The polymorphic information content ranged from 0.03 to 0.78, with an average value of 0.41, indicating a high level of polymorphism. Based on the analysis using STRUCTURE, PCoA, and UPGMA methods, the 35 chayote individuals were divided into two major clusters. Through further association analysis, 7 significantly associated SSR markers were identified, including four related to peel color and three related to spine.

**Conclusions:**

These molecular markers will contribute to the analysis of genetic diversity and genetic breeding improvement of chayote in the future.

**Supplementary Information:**

The online version contains supplementary material available at 10.1186/s12870-024-05317-9.

## Introduction

Chayote (*Sechium edule*) is a perennial climbing plant belonging to the Cucurbitaceae family. It originated in Mexico and is now also cultivated in southwest China and several coastal provinces [1]. Chayote was introduced to China during the late Ming Dynasty (1368–1644) or early Qing Dynasty (1644–1912). The exact timeline is debated, but it is generally believed that Portuguese or Spanish traders brought the plant to Asia. It quickly adapted to the subtropical and tropical climates of southern China. In China, chayote is known as ‘fó shǒu guā’. It has become a staple in southern Chinese cuisine, appreciated for its mild flavor and versatility in dishes [2]. The fruits are primarily white or green and may have a variable number of spines on their surface [3]. Chayote is a dual-use plant. Its fruit is rich in vitamins, protein, cucurbitacin, phenolic compounds, and other nutrients beneficial to humans, including trace elements like zinc and selenium [4]. Additionally, chayote has been noted for its potential benefits in treating cardiovascular diseases, lowering blood pressure, and its possible anticancer properties [5]. In recent years, chayote extracts have been explored as a source for new biodegradable plastic products in various industries [6]. Additionally, its root tubers and tendrils are edible [7]. The root tubers are rich in starch and can serve as a substitute for potatoes and sweet potatoes as a primary source of starch [8].

The precocious germination characteristics of chayote make traditional conservation methods infeasible, thereby limiting efforts to study and protect these resources [9]. Therefore, implementing field protection measures is essential for the conservation of chayote. Additionally, chayote, an underutilized crop in the Cucurbitaceae family, typically grows in poor soil conditions [10]. However, the intensification of frost, high temperatures, drought and root diseases in recent years has led to a gradual decline in wild chayote resources. In some areas, such as Mexico and Costa Rica, these resources have decreased by half [11]. The Germplasm National Bank of *Sechium edule* (BANGESe) has been established in Mexico, with most accessions originating from backyard areas. Reproductive isolation of most wild chayote materials has been influenced by consumer preferences [12]. To date, China has not carried out a systematic germplasm collection of chayote. Although chayote germplasm resources are gradually decreasing, collecting them helps maintain genetic diversity. Protecting these genetic resources is crucial for future crop improvement and ensuring food security. Thus, it is imperative to accelerate the collection of chayote germplasm resources in China [13].

Studying the genetic diversity of species aids in exploring plant evolution history, adaptation potential, and promoting genetic resource conservation. Research has shifted from enzyme-based to various DNA-based molecular markers. SSR molecular markers are considered ideal due to their co-dominance, wide distribution, high mutation rate, and high polymorphism [14]. In addition, SSR molecular markers have been widely used in Cucurbitaceae plants, such as melon [15], bitter gourd [16], cucumber [17] and bottle gourd [18]. The genetic diversity of 42 chayote from Costa Rica was analyzed using 8 isozymes, revealing 35 different polymorphic genotypes, indicating high genetic diversity [19]. With the wide application of molecular markers, 36 chayote samples from India were analyzed using 12 directed amplification of minisatellite-region DNA (DAMD) molecular markers, and 97 polymorphic bands were identified, which were finally divided into three groups due to different living environment conditions [11]. Similarly, 28 pairs of RAPD primers and 5 pairs of ISSR primers were used to analyze 74 chayote in the North Eastern Hill (NEH) region of India, and it was found that the germplasms were not grouped according to their geographical distribution. Due to the varying availability periods of chayote fruits across different regions and the fact that chayote is propagated through its fruits, traders are able to transfer popular local varieties from one planting area to another with ease [10]. Furthermore, 223 pairs of AFLP molecular markers were utilized to analyze 133 accessions of chayote resources from Mexico, revealing a high degree of genetic polymorphism between cultivated germplasm and their wild ancestors [12]. Moreover, the study showed that eight pairs of polymorphic SSR primers were screened from 20 chayote in Mexico by using 11 SSR markers developed, which will be helpful to characterize the population genetic structure of chayote varieties in Mexico and Latin America and decipher the dynamics of their genetic diversity [20]. Subsequently, the researchers used 6 pairs of primers to analyze the genetic diversity of 21 chayote samples from Japan [21].

In this study, chayote resources from the main provinces in China were collected and preserved. Additionally, we identified a rich set of Simple Sequence Repeats (SSR) molecular markers from the chayote genome data and developed reproducible polymorphic SSR markers to assess the genetic diversity and genetic structure of the collected wild germplasm resources. These findings will serve as valuable references for the conservation of chayote resources, genetic improvement, and molecular marker-assisted breeding.

## Results

### SSR analysis in chayote genome

**Fig. 1:**
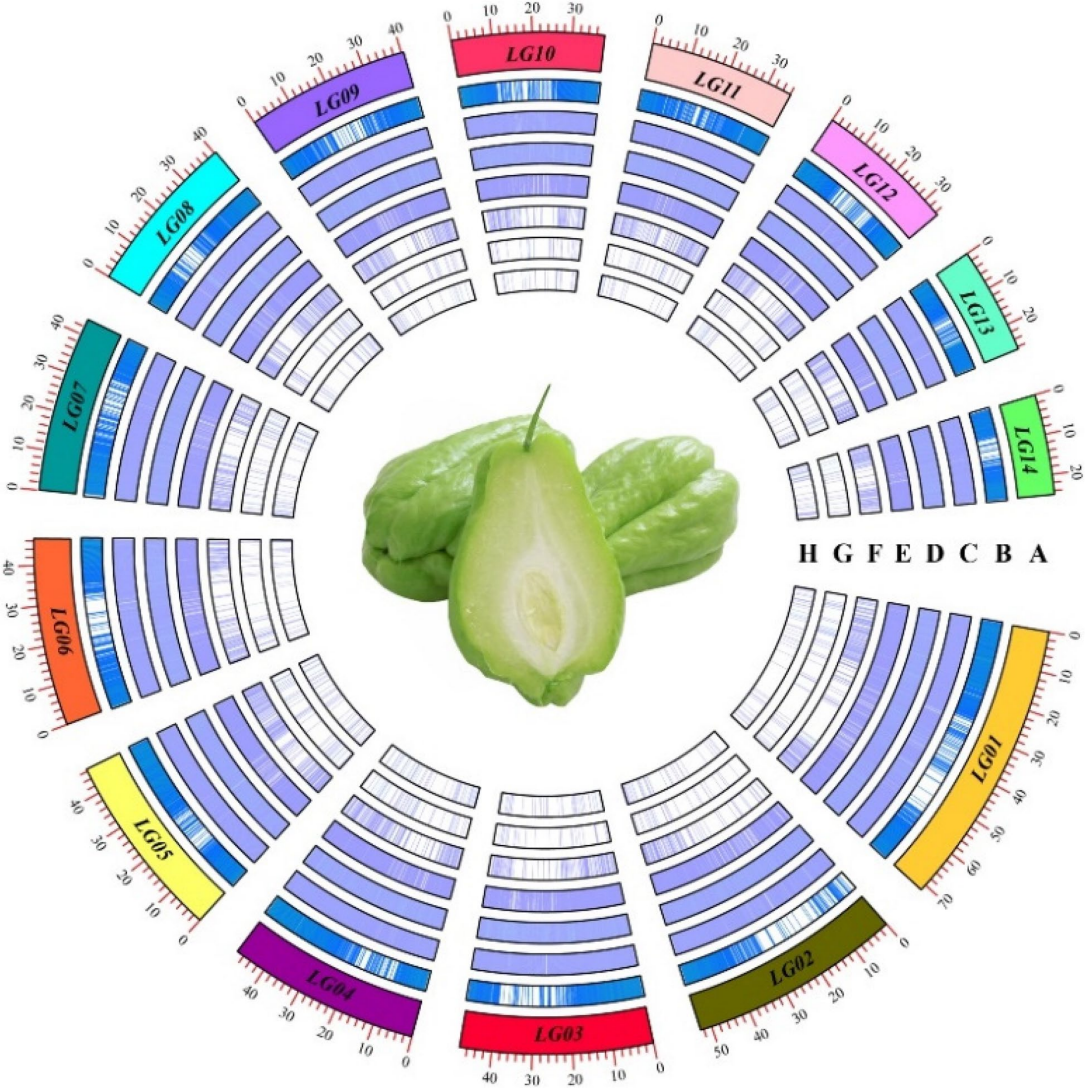
Overall view of SSR motif distribution in the chayote reference genome. **A** chayote chromosomes; **B** gene density; **C**-**H** Mono-, Di-, Tri-, Tetra-, Penta-, and Hexa- repeats, respectively

A total of 363,156 SSRs, with a relative abundance of 597.12/Mb, were identified in the genome sequence (606.42 Mb) of *S. edule*. Additionally, SSRs were found to have a total length of 5,518,289 bp and an average length of 15.2 bp. They exhibited a relative density of 9,073.52 bp/Mb. The distribution of SSR motifs with higher frequency on 14 chromosomes is summarized in Fig. 1. The information of the SSR loci’ motifs and their positions on chromosomes was also counted (Supplementary Table 2). We have designed primers for all SSR loci and successfully designed a total of 185,137 pairs of primers. We have conducted statistical analysis on the sequence and expected PCR fragment size of these primers (Supplementary Table 3).

**Fig. 2:**
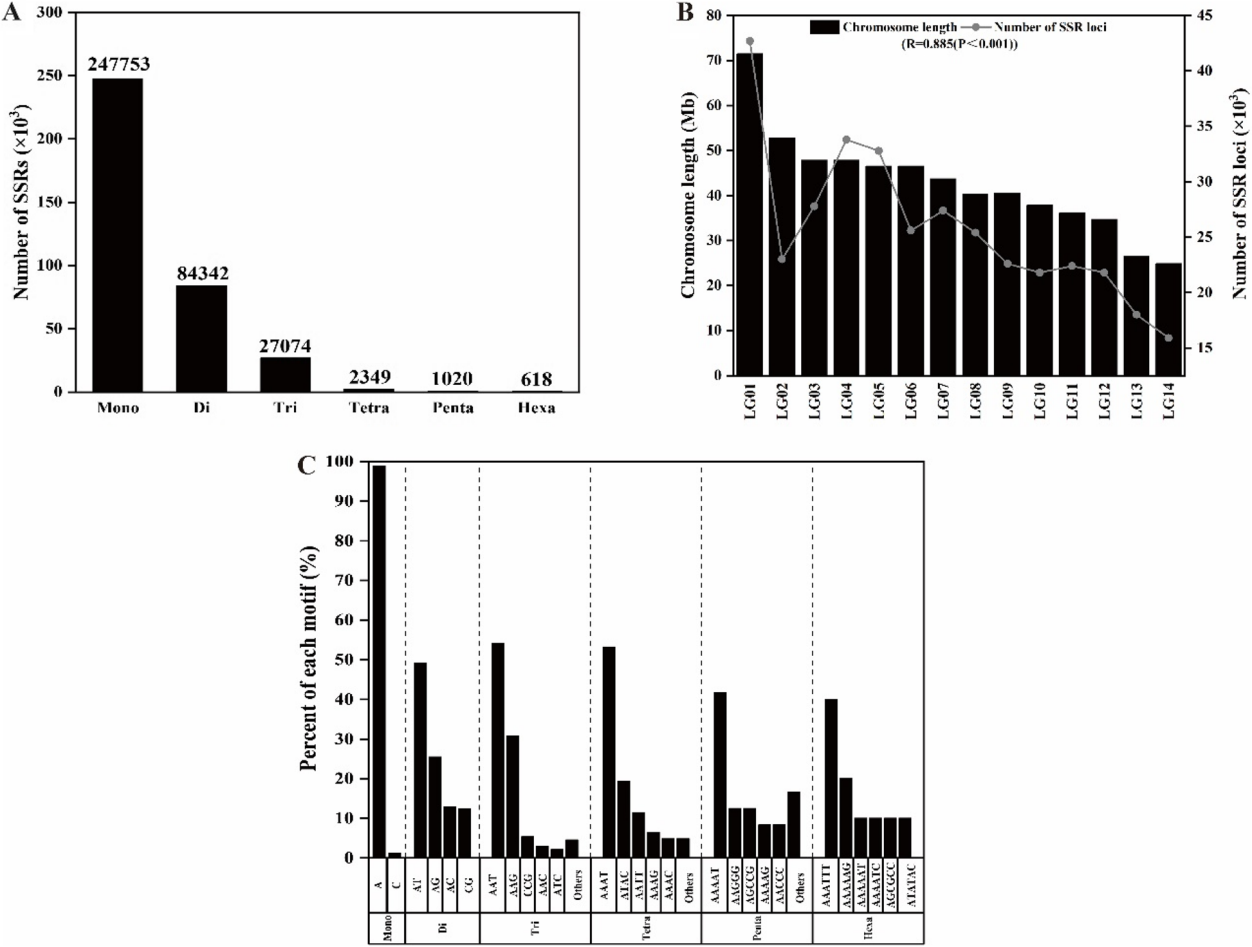
Distribution characteristics of SSR in the chayote genome. **A** Number of different SSR types; **B** Correlation between SSR number and chromosome length; **C** Frequency distribution of different SSR motif types

Among all identified SSRs, the number decreased as repeat units increased, with the largest number of repeats at 10 and 11 (Fig. 2A; Supplementary Table 4). The mononucleotide repeats were the most abundant in all identified nucleotides (247,753, 68.22%), followed by dinucleotide repeats (84,342, 23.22%) and trinucleotide repeats (27,074, 7.46%). The number and proportion of SSRs with long motifs were significantly reduced. The repeats of tetranucleotide, pentanucleotide and hexanucleotide were 2,349 (0.65%), 1,020 (0.28%) and 618 (0.17%), respectively (Fig. 2A). The number of SSRs was positively correlated with chromosome length (*r* = 0.885, *p* < 0.001), with the most SSRs on chromosome 1 and the fewest on chromosome 14 (Fig. 2B). The most abundant repetitive motif in mononucleotides was the A motif (98.8%). Among dinucleotides, AT and AG repeat motifs were the most abundant, accounting for 49.2% and 25.48%. The most abundant motifs were AAT in trinucleotides (54.16%), AAAT in tetranucleotides (53.23%), AAAAT in pentanucleotides (41.67%), and AAATTT in hexanucleotides (40%) (Fig. 2C).

### Comparative genomics analysis between chayote and other cucurbits species

**Fig. 3:**
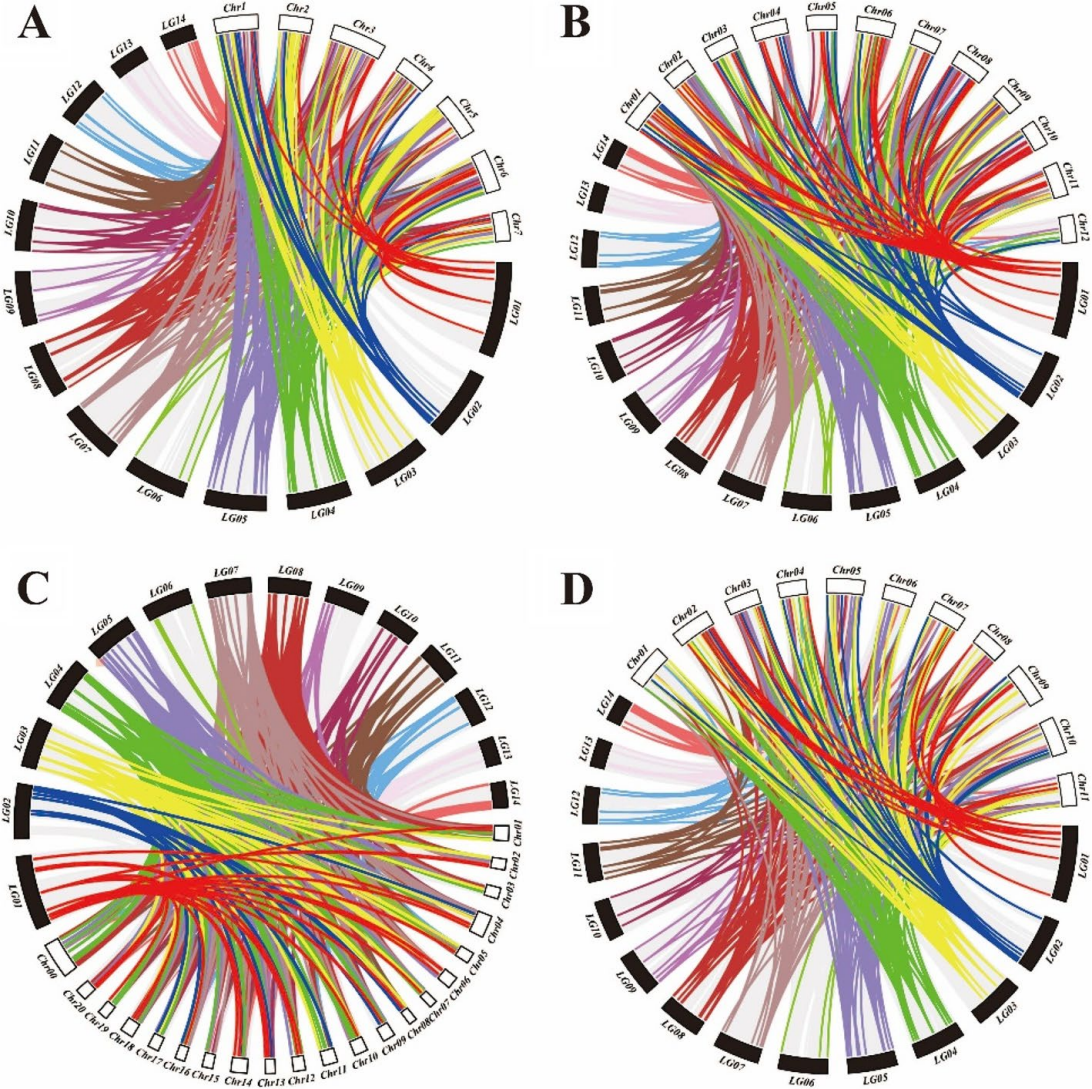
Collinearity analysis of common SSR molecular markers in chayote and cucurbits species crops. **A ***Sechium edule* with *Cucumis sativus*. **B ***Sechium edule* with *Cucumis melo*. **C ***Sechium edule* with *Cucurbita moschata*. **D ***Sechium edule* with *Citrullus lanatus*. Black represents the chromosome of chayote

We compared and analyzed the collinearity of universal SSR markers between chayote and different cucurbit species (Fig. 3). The results showed that chayote and melon had the most common SSR markers, with 1,673 (Fig. 3B), followed by cucumber with 1,264 (Fig. 3A), and pumpkin with 1,061(Fig. 3C). Watermelon had the fewest common markers, with 988 (Fig. 3D). In the collinearity between chayote and cucumber, the most common SSR markers were found on chromosome 11 of chayote, with a total of 166 interspecific SSR markers, followed by 149 SSR markers on chromosome 5. The minimum number of SSR markers found on chromosome 9 and chromosome 2 was 17 and 18, respectively (Fig. 3A; Supplementary Table 5). Among them, chromosome 6 of chayote has the least corresponding relationship with chromosomes 3, 5, and 7 of cucumber, while chromosomes 1, 3–5, 7–8, and 10–14 are collinear with all 7 chromosomes of cucumber (Supplementary Table 5). Among the collinearity between chayote and melon, the most collinearity occurred on chromosome 8 of chayote, with 163 shared SSR markers, while only 39 shared SSR markers were found on chromosome 2. The chromosomes 4, 7, and 14 of the chayote and 12 pairs of chromosomes in the melon exhibit collinearity (Supplementary Table 6). While chayote chromosomes 7 and 8 share 193 and 161 SSR markers with 20 chromosomes of pumpkin, respectively, and chayote chromosome 10 only has a minimum of 10 SSR markers with pumpkin chromosomes 5 and 9 (Supplementary Table 7). There were 159 and 20 common SSR markers on chromosomes 12 and 9 of chayote, which were the most and least common with watermelon (Supplementary Table 8).

### Analysis of genetic diversity

We randomly selected 57 pairs of SSR primers distributed on 14 chromosomes of chayote for PCR amplification. The PCR amplification results showed that 48 out of 57 primer pairs amplified bands, of which 42 primer pairs had polymorphic bands. These 42 primer pairs detected a total of 153 alleles in 35 chayote resources, with an average amplification of 3.64 loci per primer pair (Supplementary Table 9). The observed variation in the number of alleles (Na) ranges from 2 to 9, with SSR61 having the highest Na value of 9. The variation range of effective allele number (Ne) ranged between 1.03 to 5.23, with an average value of 2.289. The variation ranges of observed heterozygosity (Ho) and expected heterozygosity (He) were 0.17 to 0.97 and 0.03 to 0.83, respectively. In addition, the Shannon Information Index (I) ranged from 0.08 to 1.80, with a mean of 0.85. The Nei's gene diversity index (H) ranged from 0.03 to 0.81, with a mean of 0.48. The variation range of polymorphism information content (PIC) was 0.03 to 0.78, with a mean of 0.41. Among them, 14 SSR primers were highly polymorphic (PIC ≥ 0.5), 20 SSR primers were moderately polymorphic (0.25 < PIC < 0.5), and 8 primers were low polymorphic (PIC ≤ 0.25) (Table 1).

**Table 1:** Genetic diversity parameters of 42 SSR markers in 35 chayote materials

Primer name	Na	Ne	Ho	He	I	H	PIC
SSR61	9.00	4.39	0.20	0.80	1.78	0.77	0.75
SSR114	8.00	3.37	0.29	0.71	1.46	0.70	0.66
SSR139	3.00	2.01	0.49	0.51	0.85	0.50	0.44
SSR613	5.00	2.57	0.38	0.62	1.18	0.61	0.56
SSR42749	5.00	2.43	0.40	0.60	1.13	0.59	0.54
SSR53846	2.00	1.99	0.49	0.51	0.69	0.50	0.37
SSR65750	2.00	1.23	0.81	0.19	0.33	0.18	0.17
SSR69944	3.00	2.14	0.46	0.54	0.82	0.53	0.42
SSR73677	4.00	2.12	0.46	0.54	0.93	0.53	0.46
SSR76953	2.00	1.92	0.51	0.49	0.67	0.48	0.36
SSR93286	2.00	1.98	0.50	0.50	0.69	0.50	0.37
SSR108420	3.00	1.82	0.54	0.46	0.74	0.45	0.38
SSR111053	2.00	1.99	0.49	0.51	0.69	0.50	0.37
SSR151039	4.00	3.08	0.31	0.69	1.25	0.68	0.63
SSR151215	4.00	1.49	0.66	0.34	0.66	0.33	0.31
SSR159645	2.00	2.00	0.49	0.51	0.69	0.50	0.37
SSR160502	2.00	2.00	0.49	0.51	0.69	0.50	0.37
SSR161040	4.00	2.18	0.45	0.55	0.87	0.54	0.44
SSR185330	2.00	1.06	0.94	0.06	0.14	0.06	0.06
SSR212641	2.00	2.00	0.49	0.51	0.69	0.50	0.37
SSR216561	3.00	2.98	0.33	0.67	1.10	0.66	0.59
SSR243677	2.00	1.08	0.92	0.08	0.17	0.08	0.07
SSR243820	2.00	1.99	0.49	0.51	0.69	0.50	0.37
SSR247694	2.00	1.03	0.97	0.03	0.08	0.03	0.03
SSR261825	2.00	1.13	0.88	0.12	0.23	0.12	0.11
SSR267344	2.00	1.06	0.94	0.06	0.13	0.06	0.05
SSR267366	7.00	4.81	0.20	0.80	1.67	0.79	0.76
SSR282418	7.00	4.38	0.21	0.79	1.64	0.77	0.74
SSR292741	3.00	1.14	0.88	0.12	0.28	0.12	0.12
SSR293332	2.00	2.00	0.49	0.51	0.69	0.50	0.37
SSR304785	5.00	1.78	0.55	0.45	0.92	0.44	0.42
SSR307134	2.00	1.42	0.70	0.30	0.47	0.30	0.25
SSR313484	5.00	4.30	0.22	0.78	1.53	0.77	0.73
SSR318481	8.00	5.23	0.17	0.83	1.80	0.81	0.78
SSR326475	7.00	2.83	0.34	0.66	1.26	0.65	0.58
SSR327195	3.00	1.81	0.55	0.45	0.69	0.45	0.36
SSR327794	4.00	2.63	0.37	0.63	1.13	0.62	0.56
SSR332709	4.00	2.25	0.44	0.57	0.91	0.56	0.46
SSR345052	2.00	1.06	0.94	0.06	0.13	0.06	0.06
SSR349227	4.00	2.94	0.33	0.67	1.19	0.66	0.60
SSR353503	2.00	1.84	0.54	0.46	0.65	0.46	0.35
SSR353506	5.00	2.65	0.37	0.63	1.16	0.62	0.55
Mean	3.64	2.29	0.52	0.48	0.85	0.48	0.41

**Fig. 4:**
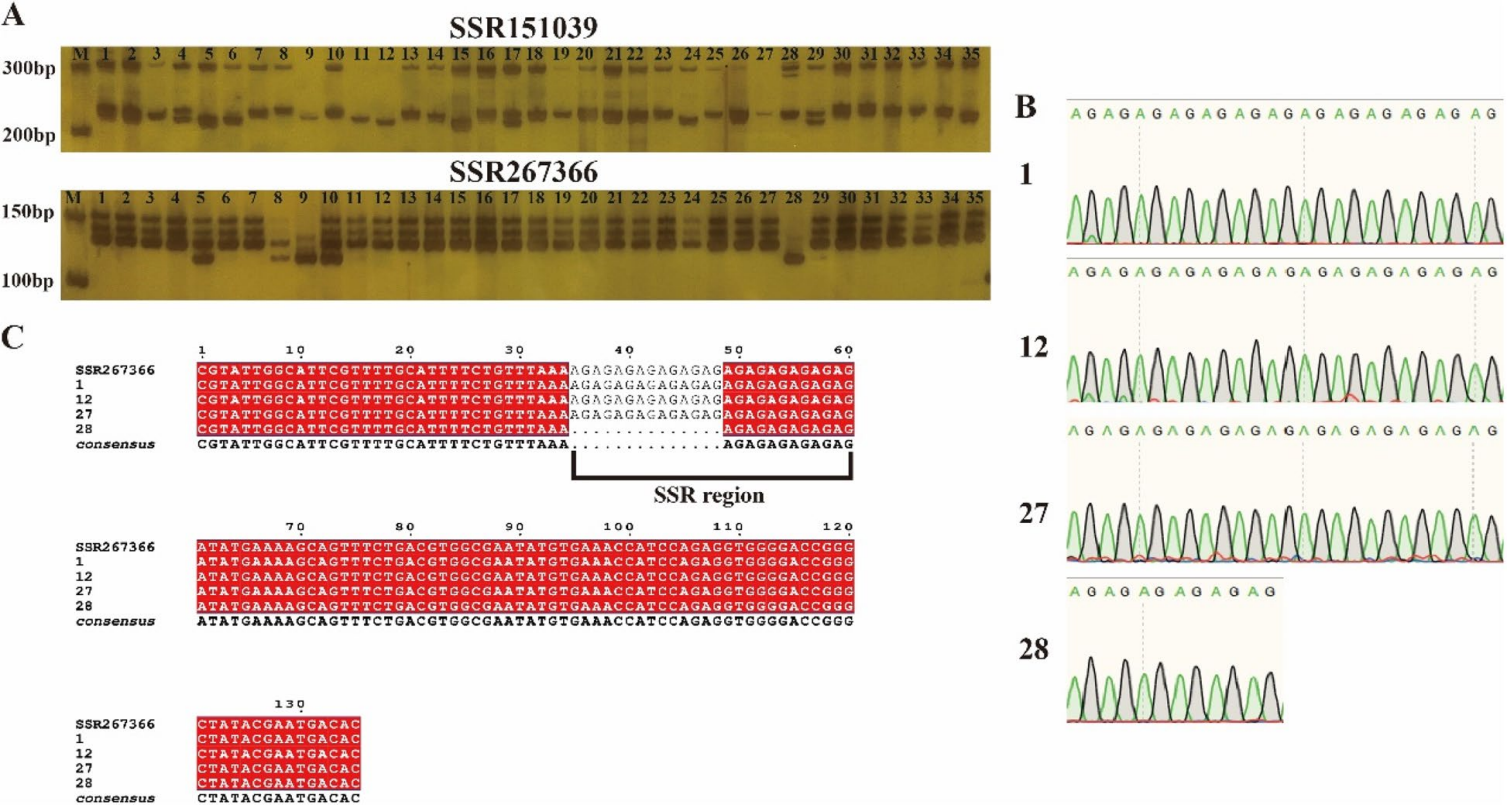
Representative SSR polyacrylamide gel electrophoresis and sanger sequencing. **A** Results of PCR amplification profile of 35 chayote germplasm resources using SSR151039 and SSR267366. M: DNA Ladder; 1 to 35: materials of the 35 chayote individuals listed in Table 3. **B** Sequencing peak map of SSR267366 marker in chayote with material numbers 1, 12, 27 and 28. C: Multiple sequence alignment results of SSR267366 marker in chayote with material numbers 1, 12, 27 and 28. SSR repeat motifs were marked with black lines

In order to determine whether the difference in the bands of polyacrylamide gel electrophoresis results is due to the difference in the number of SSR repeats. We selected SSR267366 molecular marker and randomly recovered the target bands of PCR products from four materials (No. 1, 12, 27 and 28) for sequencing. The results showed that No. 1, No. 12 and No. 27 had the same number of AG repeat motifs, while No. 28 had fewer repeat motifs than materials 1, 12 and 27 (Fig. 4B). We observed the same results on polyacrylamide gel (Fig. 4A). Multiple sequence alignment further confirmed that all four materials contained conserved AG motifs (35–60 bp), and the differences in bands were due to differences in the number of SSR motif repeats among different chayote resources (Fig. 4C).

### Cluster analysis based on SSR markers

**Fig. 5:**
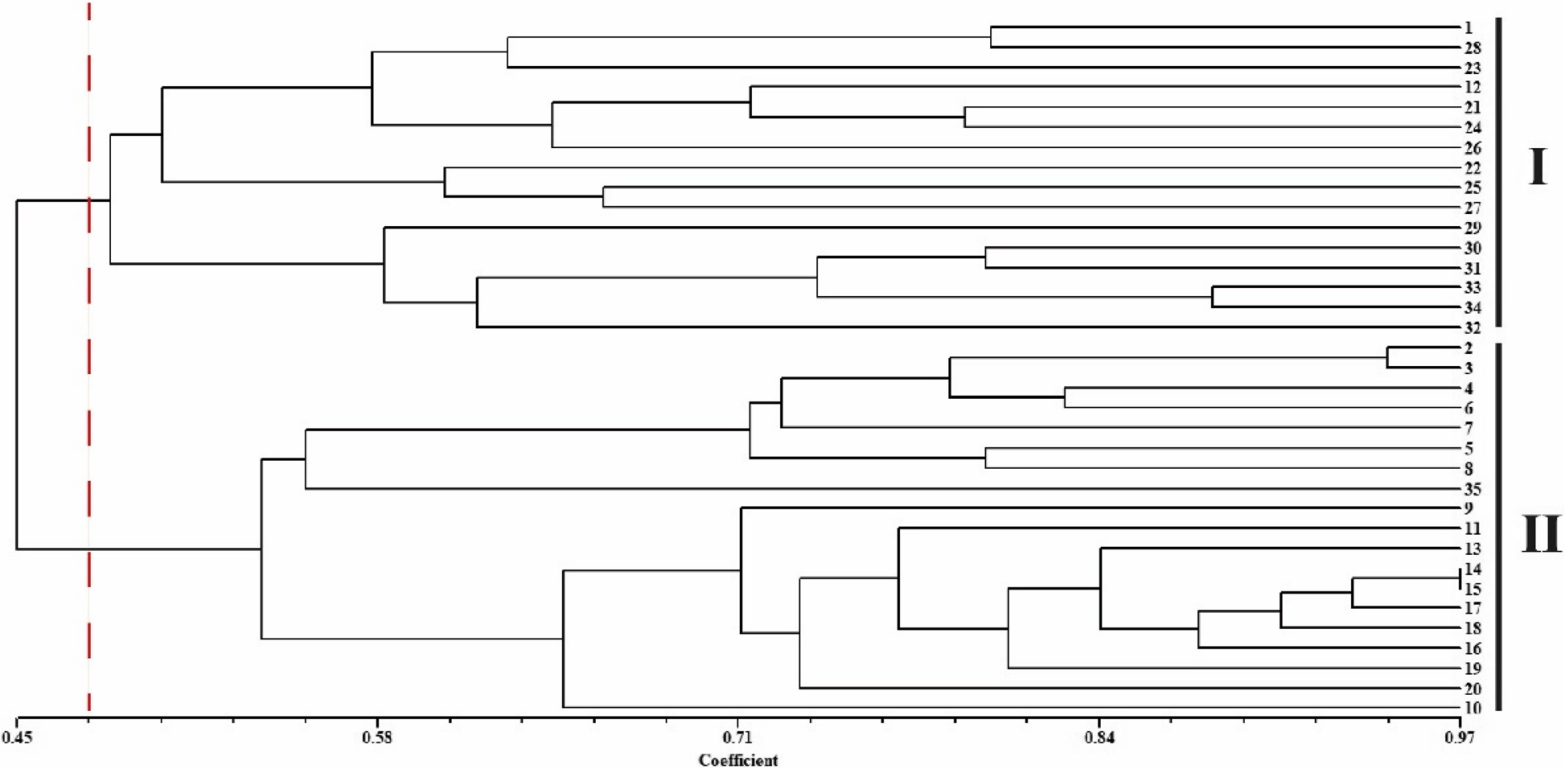
Dendrogram of 35 chayote individuals based on UPGMA

The genetic similarity coefficient of 35 germplasms was calculated based on SSR data, and UPGMA cluster analysis was performed using genetic distance (Fig. 5). The genetic similarity coefficients ranged from 0.45 to 0.97, with a variance of 0.52. Materials numbered 14 and 15 had the highest similarity coefficient of 0.97, indicating the closest genetic relationship. At a genetic coefficient of 0.476, the 35 materials were divided into two major clusters. The first cluster was divided into two sub-clusters. The first sub-cluster included No.12 chayote from Hubei Province, and the remaining 9 materials from Sichuan Province. Additionally, materials numbered 22, 25, and 27, all with white fruit skin, formed a separate small cluster. The second sub-cluster included 6 chayote materials (numbered 29–34) from Yunnan Province. The remaining 19 materials formed the second major cluster.

### Principal coordinate analysis

**Fig. 6:**
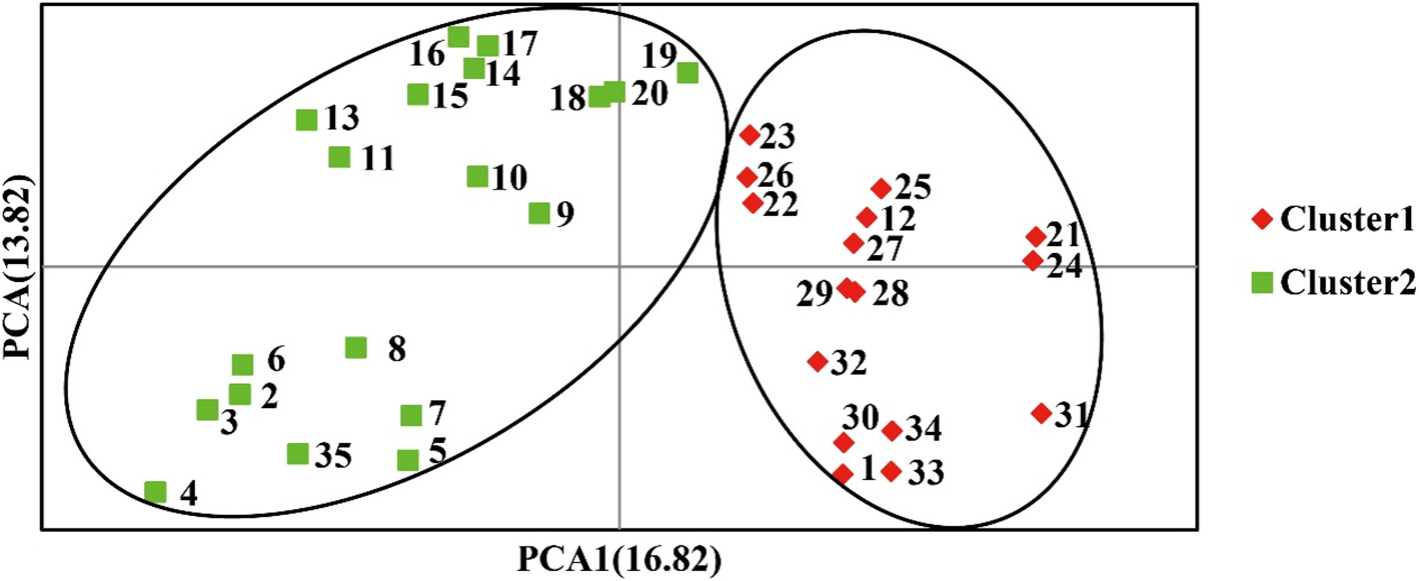
Principal coordinates analysis of 35 chayote based on SSR data

Principal coordinate analysis (PCoA) based on genetic distance was performed to observe the genetic relationship among 35 chayote genotypes (Fig. 6). The first and second axes explained 16.82% and 13.82% of the variation, respectively. The 35 chayote samples were divided into two clusters. The 35 chayote samples were divided into two clusters. The first cluster included 16 samples (1, 12, and 21–34), while the second cluster included 19 samples (2–11, 13–20, and 35).

### Population structure analysis

**Fig. 7:**
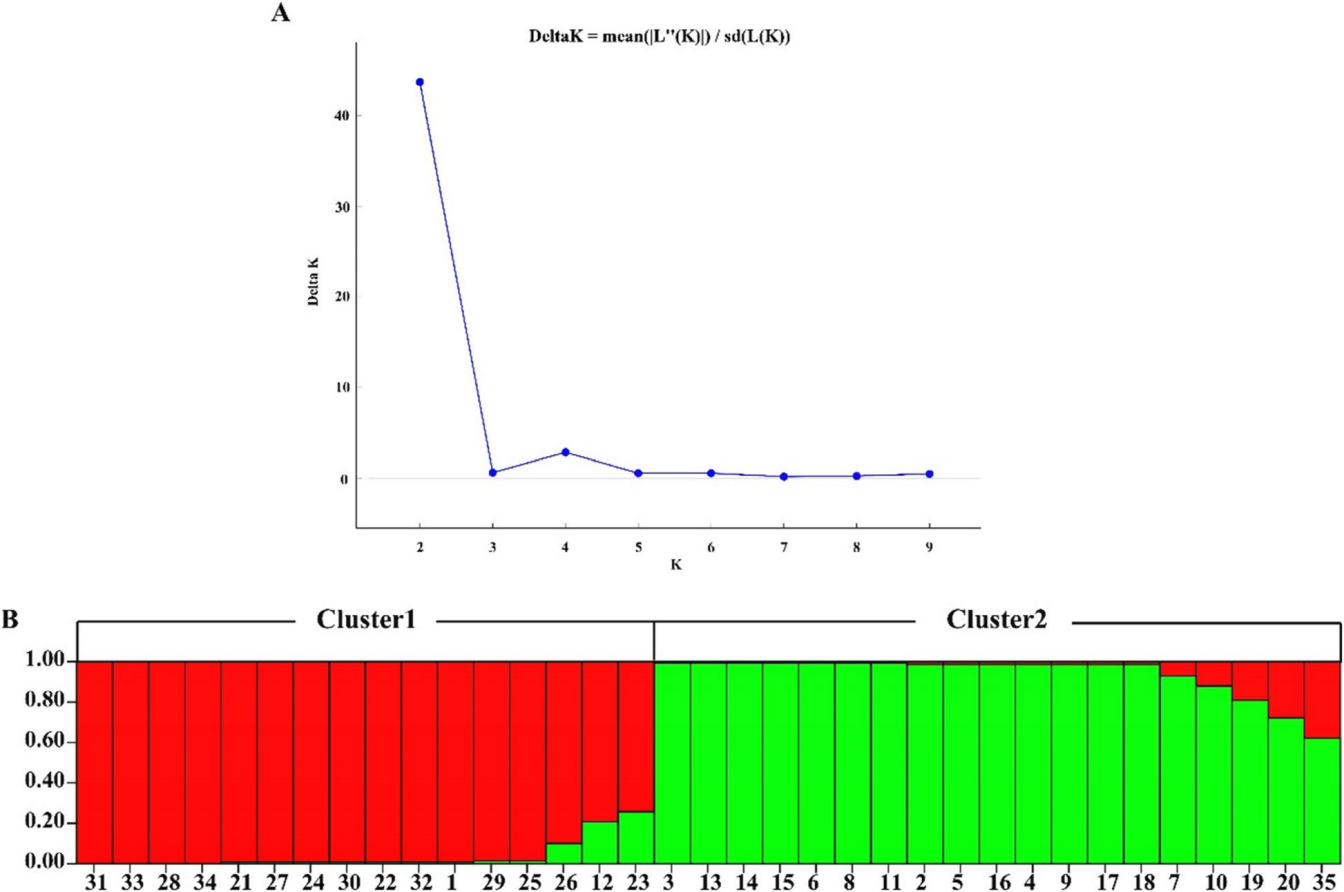
The population structure of 35 chayote germplasms. **A** Line chart of K value and ΔK value based on SSR markers; **B** Population structure analysis of 35 chayote germplasm materials (K = 2)

Structure software was used to infer population structure of the 35 chayote samples (Fig. 7). The results show that when K = 2, Δ K has a clear highest peak. Therefore, the 35 materials were divided into two main clusters. The population structure classification results were consistent with the PCoA classification results. Among the 16 materials in the first cluster, 9 were sourced from Sichuan Province (numbered 1 and 21–28), 6 were sourced from Yunnan Province (numbered 29–34), and 1 was sourced from Hubei Province (numbered 12). The 19 materials for the second cluster were sourced from 11 provinces, respectively.

### Trait (peel color and spine)—SSR association analysis

We conducted an association analysis between fruit peel color and spine with SSR molecular markers by taking Q (population structure) and K (relative kinship) as covariables using MLM and GLM models (Table 2). GLM model showed that three SSR markers were significantly associated with peel color (*P* < 0.001) and explained 44.71–46.05% of phenotypic variation. SSR108420, SSR185330 and SSR247694 were significantly associated with spine (*P* < 0.05), which explained the phenotypic variation of 11.94, 16.08 and 11.75% respectively. In MLM model, SSR332709 was significantly associated with peel color (*P* < 0.05), which explained 23.33% phenotypic variation. SSR108420 and SSR185330 also detected significant associated with spine in MLM model (*P* < 0.05).

**Table 2:** Results of association analysis between SSR markers and fruit traits

Trait	GLM			MLM		
	**Marker**	*P*-value	**PVE (%)**	**Marker**	*P*-value	**PVE (%)**
Peel color	SSR151215	1.69E-05	45.10%	SSR332709	2.87E-02	23.33%
	SSR185263	1.20E-05	46.05%			
	SSR161040	2.62E-06	44.71%			
Spine	SSR108420	2.55E-02	11.94%	SSR108420	1.98E-02	17.65%
	SSR185330	3.33E-02	16.08%	SSR185330	3.34E-02	29.07%
	SSR247694	2.69E-02	11.75%			

## Discussion

Chayote is an edible and medicinal plant distributed in southwest China. Currently, the planting area of chayote is gradually decreasing, leading to the loss of some germplasm resources. It is also an important but underutilized Cucurbitaceae crop [2]. Therefore, collecting and analyzing chayote germplasm resources is crucial for their protection and utilization. This study collected germplasm resources from the main chayote growing areas in China, which are the most reported current germplasm resources of chayote. These germplasm resources will provide considerable opportunities for genetic improvement and breeding in the future.

SSRs are reliable genetic markers for genetic diversity research and breeding. Few studies have developed and applied SSR markers to chayote. Initially, only 11 SSR markers were developed from AC and GA-enriched genomic libraries [20]. Subsequently, six SSR markers were used to analyze the genetic diversity of chayote [21]. With the release of chayote genome, it is possible to develop SSRs at the whole genome level [22]. In this study, a total of 363,156 SSRs were identified in chayote, more than 296,360 SSRs in watermelon (97,103), 254,243 SSRs in melon (DHL92), and 188,091 SSRs in bitter gourd (Dali-11). [16]. At the same time, the SSR marker density of chayote is 597.12 loci/Mb, which is lower than that of bitter gourd (639.73 loci/Mb), cucumber (658.72 loci/Mb), melon (624.78 loci/Mb) and watermelon (834.24 loci/Mb) [16]. This situation may be due to the difference of species, as well as the different SSR identification software and parameters used by developers. The parameters set by SSR of these Cucurbitaceae species are that trinucleotide (Tri-), tetranucleotide (Tetra-), pentanucleotide (Penta-) and hexanucleotide (Hexa-) are repeated four times, while the standard set in this study is at least 5 times. Previous studies have shown that the more repetitions, the higher the polymorphism [23]. We identified 124,177, 221,986, 260,094, and 163,710 SSR motifs in the genomes of cucumber, melon, pumpkin, and watermelon, respectively, using the same parameters. All of these numbers are lower than the number of SSR motifs found in the chayote genome. The SSR marker density in chayote is higher than that in cucumber (549.03 loci/Mb) but lower than that in melon (620.53 loci/Mb), pumpkin (722.88 loci/Mb), and watermelon (622.53 loci/Mb). This situation may be attributed to differences in genome size directly affecting marker density, uneven distribution of SSR markers within the genome, variations in the number and types of repeat sequences among different plants, evolutionary and breeding histories influencing SSR marker distribution, and varying quality of genome sequencing and annotation across crops.

Previous studies have shown that SSR will be enriched at the end of chromosome [24], and SSR motifs of A and AT account for a higher proportion in mononucleotide and dinucleotide [25]. These characteristics can be explained by the fact that there are fewer genes in the centromere region and the downstream genes are rich in poly(A) structure [26]. In this study, the proportion of mononucleotide and dinucleotide in chayote was up to 91.44%, which was consistent with the reported Cucurbitaceae crops [16]. Notably, the number of SSR motifs of A and AT is much higher than that of C and GC. This is consistent with many reported plants, such as *Arabidopsis thaliana* [27], *Akebia trifoliata* [28], *Monochasma savatieri* [29]. This may be attributed to the long evolutionary history of dicotyledons and the higher frequency of AT motifs [30]. Moreover, we conducted comparative genomic analysis of chayote and cucurbit species to explore the collinearity of shared SSR markers among different species. The collinearity results revealed that the genomes of chayote, cucumber, melon, pumpkin, and watermelon were relatively conserved. Comparative genomics has important value in studying the evolutionary process between different cucurbit species.

Studying the genetic diversity of species is not only helpful to explore its adaptability and evolution history, but also provides an important theoretical basis for the protection and utilization of species [31]. In this study, 48 pairs of polymorphic primers were screened from 57 pairs of SSR primers designed in the genome of chayote. The average values of observed heterozygosity and expected heterozygosity among 35 chayote germplasm were 0.52 and 0.48, respectively, and the average value of Shannon information index was 0.85. Previous studies showed that the average values of Ho and He in 21 samples of chayote were 0.29 and 0.41 respectively, and the average value of Shannon information index was 0.61 [21]. The results showed that the genetic differentiation and polymorphism of chayote germplasm resources collected in this study were high. Moreover, the observed heterozygosity is slightly higher than the expected heterozygosity, indicating that there is a surplus of homozygotes among chayote resources. The level of genetic diversity of species is closely related to their mating system, and the observed heterozygosity of mainly outcrossing species is obviously less than the expected heterozygosity [32]. Although chayote is an insect-borne plant, natural geographical barriers such as mountains and lakes lead to inbreeding among individuals of chayote, resulting in too many homozygotes. The high expected heterozygosity among chayote germplasm resources indicates that these resources are rich in genetic diversity, which can be properly protected.

Current studies indicate that factors influencing species genetic diversity include reproductive mode, natural selection, gene flow, genetic drift, and gene mutation [33]. Therefore, genetic diversity analysis is crucial for accurately understanding the genetic relationships between germplasm [34]. In this study, 35 chayote germplasm resources were divided into two clusters based on the three kinds of analysis of Structure, PCoA, and UPGMA. UPGMA clustering analysis divided 35 materials into two clusters, with the first cluster mainly including materials from Yunnan and Sichuan provinces. Notably, three portions of chayote with white peel color were grouped into a small group. Materials from similar sources were also grouped separately into a small group. The structure analysis showed that the materials in Sichuan and Yunnan were divided into one cluster, and the remaining cluster mainly included coastal provinces and some provinces in the central region. The chayote materials showed a certain regional distribution pattern. PCoA analysis once again obtained the same results. In general, geographical isolation may be the main factor affecting the genetic differentiation of chayote population, leading to a high level of genetic differentiation and variation of the species.

Peel color and spine are important agronomic and commercial characteristics that affect the appearance, quality, and value of fruits. They also play a crucial role in plant defense against biotic and abiotic stresses [35]. Chayote fruit is mainly white or green, and the presence or absence of spines is also an important characteristic [4]. Association analysis can combine polymorphic allelic variation sites with target phenotypic data, and can directly identify gene sites closely related to phenotypic variation. However, the accuracy of correlation analysis results is influenced by many factors [36]. The population structure is an important factor affecting the results of association analysis. The mixing of subgroups makes the LD intensity of the whole population increase, which may lead to the association between unlinked gene polymorphism sites and traits, thus obtaining false positive results. Therefore, the simpler the population structure, the more reliable the association analysis results [37]. In this study, 35 chayote materials can be divided into two clusters, and the population structure is relatively simple, which ensures the accuracy of the correlation analysis results to some extent. Moreover, GLM and MLM models are selected for correlation analysis to ensure the reliability of the correlation results. A total of 4 SSR loci were detected which were significantly associated with peel color and 3 with spine, among which SSR108420 and SSR185330 were detected in both GLM and MLM models. At present, the research on correlation analysis of chayote has not been reported, so the SSR markers significantly correlated in this study need further experiments to verify the accuracy of the results.

## Conclusion

In this study, 363,156 SSR motifs were found at the genome level of chayote for the first time. Among them, 48 highly polymorphic SSR markers divided 35 chayote resources into two clusters. Geographic isolation plays an important role in genetic differentiation between populations. Moreover, 4 and 3 SSR markers were significantly associated with peel color and spine traits. These SSR markers and population clustering results will play an important role in the future genetic improvement and breeding plans of chayote.

## Materials and methods

### Plant materials and DNA extraction

**Fig. 8:**
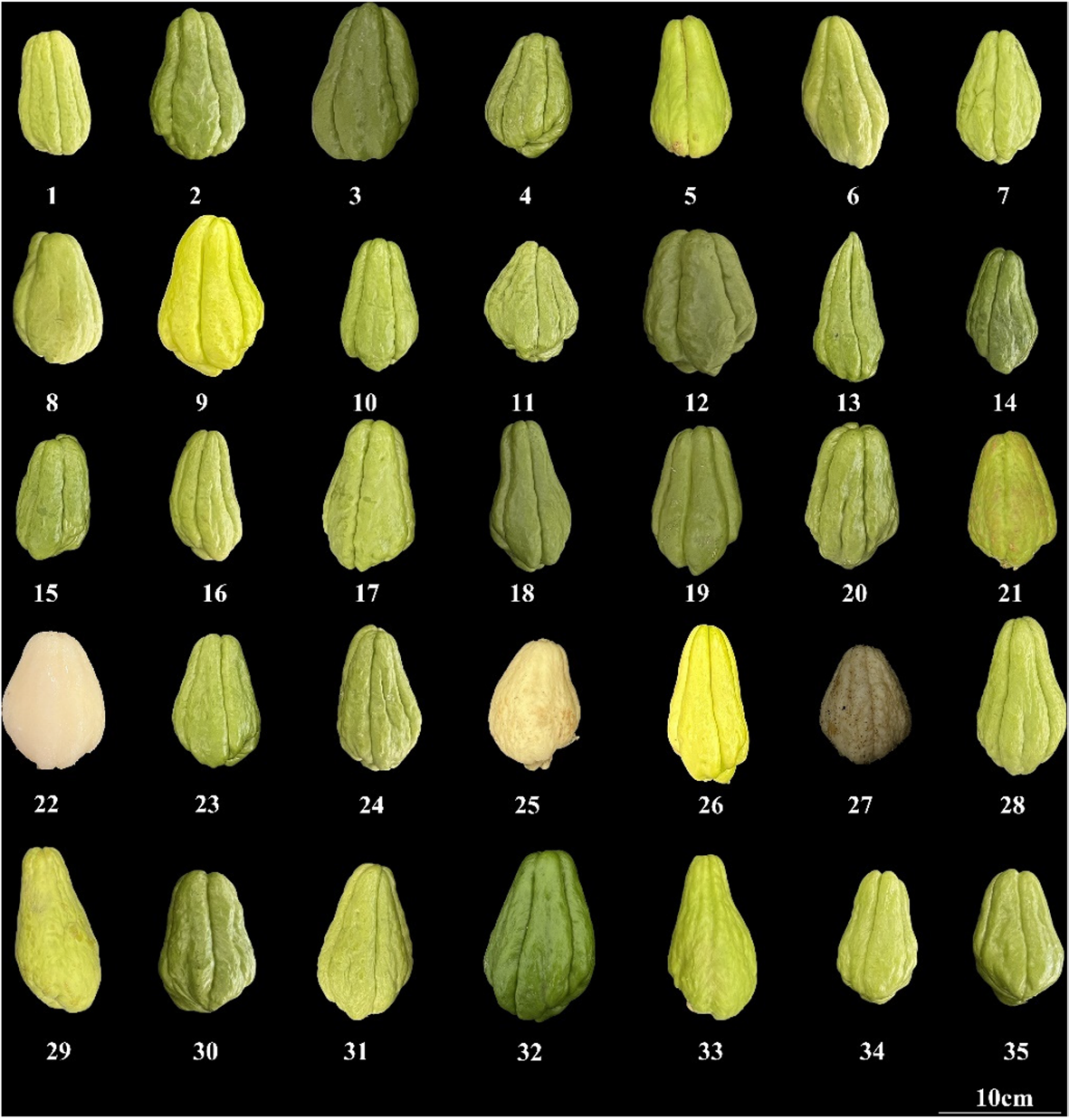
Phenotypic photos of 35 chayote fruits germplasm resources

A total of 35 chayote genotypes collected from 14 provinces in China were used in this study (Table 3, Fig. 8). There were 9 accessions from different regions in Sichuan Province, 6 from Yunnan Province, 3 from Guizhou, Guangxi, and Jiangsu provinces, 2 from Anhui and Shandong provinces, and 1 from Fujian, Guangdong, Hunan, Hubei, Shaanxi, Shanghai, and Zhejiang provinces. All materials were planted in the modern agricultural research and development base of Sichuan Agricultural University (Chongzhou, Sichuan). The leaves of three samples from each material were mixed in equal quantities and stored at -80℃ for DNA extraction. Genomic DNA was extracted by CTAB method [38]. The quality and concentration of DNA were determined by Nanodrop One ultra-micro ultraviolet spectrophotometer, and the final concentration of DNA was adjusted to 100 ng/μL for subsequent experiments.

**Table 3:** Details and sources of the 35 chayote samples analyzed in this study

Number	Origin	Sample types	Longitude	Latitude	Fruit skin color	Spine
1	Sichuan agricultural university	Cultivars (Chuanya No.1)	103.862	30.705	light green	very low
2	Fuyang, Anhui	Landraces	115.820	32.897	dark green	none
3	Suzhou, Anhui	Landraces	116.984	33.634	dark green	none
4	Fuzhou, Fujian	Landraces	119.273	26.048	dark green	none
5	Guangzhou, Guangdong	Landraces	113.281	23.125	green	none
6	Laibin, Guangxi	Landraces	109.230	23.734	green	none
7	Liuzhou, Guangxi	Landraces	109.412	24.315	green	very low
8	Yulin, Guangxi	Landraces	110.051	22.580	green	none
9	Anshun, Guizhou	Landraces	105.932	26.246	green	none
10	Liupanshui, Guizhou	Landraces	104.847	26.585	green	none
11	Qiannan, Guizhou	Landraces	107.522	26.254	green	none
12	Enshi, Hubei	Landraces	109.488	30.272	dark green	none
13	Huaihua, Hunan	Landraces	109.978	27.550	dark green	very low
14	Huaian, Jiangsu	Landraces	119.021	33.598	dark green	none
15	Suqian, Jiangsu	Landraces	118.293	33.945	dark green	none
16	Xuzhou, Jiangsu	Landraces	117.184	34.193	green	none
17	Rizhao, Shandong	Landraces	119.461	35.429	green	none
18	Weifang, Shandong	Landraces	119.166	36.655	dark green	none
19	Xian, Shanxi	Landraces	108.948	34.263	dark green	none
20	Shanghai	Landraces	121.473	31.232	dark green	none
21	Chengdu, Sichuan	Landraces	103.856	30.681	green	none
22	Deyang, Sichuan	Landraces	104.399	31.128	white	none
23	Leshan, Sichuan	Landraces	103.079	29.244	green	very low
24	Liangshan, Sichuan	Landraces	102.267	27.882	green	none
25	Jintang, Sichuan	Landraces	104.412	30.862	white	none
26	Jintang, Sichuan	Landraces	104.412	30.862	light green	none
27	Neijiang, Sichuan	Landraces	105.075	29.593	white	very low
28	Yaan, Sichuan	Landraces	103.110	30.070	light green	none
29	Baoshan, Yunnan	Landraces	99.166	25.121	green	none
30	Dehong, Yunnan	Landraces	98.585	24.432	dark green	none
31	Honghe, Yunnan	Landraces	103.376	23.364	green	none
32	Kunming, Yunnan	Landraces	102.821	24.886	dark green	none
33	Qujing, Yunann	Landraces	103.822	25.602	green	none
34	Wenshan, Yunnan	Landraces	104.215	23.398	green	none
35	Taizhou, Zhejiang	Landraces	121.43	28.66	dark green	none

### Genome-wide SSR mining and primer development

Chayote genome data was sourced from the CuGenDBv2 genome website (http://cucurbitgenomics.org/v2/). Whole-genome SSRs of chayote were detected using Krait software [39], with minimum repeat numbers of ten for mononucleotides (Mono-) and six for dinucleotides (Di-). Five repeats were set for trinucleotides (Tri-), tetranucleotides (Tetra-), pentanucleotides (Penta-), and hexanucleotides (Hexa-). 57 SSR markers distributed across 14 chromosomes of the chayote genome were randomly selected, and primers were designed using Primer 5.0 software (Supplementary Table 1). The specific parameters were: PCR product length of 100–250 bp, primer length of 18–24 bp, annealing temperature of 50–60℃, and GC content of 40–60% [40].

### Comparative genome between chayote and other cucurbits species

To analyze the collinearity of the developed SSR markers in the genomes of Cucurbitaceae species, we conducted a BLAST search on the genomes of cucumbers, cantaloupes, pumpkins, and watermelons in the Cucurbitaceae family using all identified repetitive motifs from the chayote genome, with an expected value (E-value) of 10. We allowed primers with a maximum of five nucleotide mismatches at the 5' end, but no mismatches at the 3' end, and a total matching homology of at least 90%. We removed the duplicated parts and the comparison on the scaffolds from the comparison results. Finally, we analyzed the collinearity of SSR markers between species using Circos v0.55 software.

### PCR amplification and polyacrylamide gel electrophoresis

We used a 20 μL reaction mixture for PCR amplification, specifically including 10 μL 2 × SanTaq PCR Mix (Shanghai Biotech. Shanghai, China), 1 μL primer and genomic DNA, 7 μL ddH_2_O. The PCR amplification reaction procedure is as follows: 94℃ pre-denaturation for 5 min; The following 35 cycles: 94℃ denaturation for 30 s, 55℃ annealing for 30 s, and 72 ℃ extension for 30 s; Finally, extension at 72℃ for 10 min. PCR products were detected by using 10% polyacrylamide gel containing 1 × TBE buffer, and electrophoresis was carried out at 180 V for 2 h. After electrophoresis, silver staining was performed and photos were recorded [41].

### Cloning and sequencing of PCR products

In order to confirm whether the difference in the banding pattern is caused by the difference in the number of SSR repeats, we randomly selected an SSR marker to randomly cut the amplified PCR products of four chayote resources from the 10% polyacrylamide gel, and recycled the target DNA through the EZ-10 Spin Column DNA PAGE Gel Extraction Kit (Sangon Biotech, China), then connected it to the clone T vector to transform E. coli, and finally selected a single clone for Sanger sequencing. Multi-sequence alignment was carried out by using MEGA software to confirm the difference of SSR motifs numbers.

### Data analysis

The basic parameters of each SSR marker were counted by Power Marker (v3.25) software, including observed allele number (Na), effective allele number (Ne), observed heterozygosity (Ho), expected heterozygosity (He), Shannon's information index (I), Nei's gene diversity index (H) and polymorphism information index (PIC) [42]. Principal component analysis was conducted using GenAIEx (v6.51b) software. Structure (v2.3.4) software was used to analyze the population genetic structure. Set the ancestral value from 1 to 10, and assume that all loci are independent. Set the initial uncounted iteration of MCMC to 10,000 times, and then set the uncounted iteration to 100,000 times and 10 repetitions. Determine the best k value by using the online evaluation software Structure Harvester (Web v0.6.94) [43]. The software NTsys-pc (v2.10 e) was used for cluster analysis among varieties based on unweighted pair group with arithmetic average (UPGMA). The relationship between SSR markers and the phenotypic traits of chayote peel color and spine was analyzed by using the mixed linear model (MLM) and general linear model (GLM) of Tassel (v2.1) software [44].

### Supplementary Information

Supplementary Material 1.

Supplementary Material 2.

Supplementary Material 3.

Supplementary Material 4.

Supplementary Material 5.

Supplementary Material 6.

Supplementary Material 7.

Supplementary Material 8.

Supplementary Material 9.

## Data Availability

All data generated or analyzed during this study are included in the manuscript and its Supplemental Table 1 to Supplemental Table 8. The materials used during the current study are available from the corresponding authors. The SSR makers of chayote were deposited and are available at https://github.com/shaobocheng/SSR-maker.
